# VEGF Profile in Early Undifferentiated Arthritis Cohort

**DOI:** 10.3390/medicina58060833

**Published:** 2022-06-20

**Authors:** Regina Sakalyte, Loreta Bagdonaite, Sigita Stropuviene, Sarune Naktinyte, Algirdas Venalis

**Affiliations:** 1 The Clinic of Rheumatology, Traumatology Orthopaedics and Reconstructive Surgery, Institute of Clinical Medicine, Faculty of Medicine, Vilnius University, M. K. Čiurlionio str. 21, 03101 Vilnius, Lithuania; sigita.stropuviene@santa.lt (S.S.); algirdas.venalis@imcentras.lt (A.V.); 2 State Research Institute Centre for Innovative Medicine, Santariškių g. 5, 08406 Vilnius, Lithuania; 3 Department of Physiology, Biochemistry, Microbiology and Laboratory Medicine, Institute of Biomedical Science, Faculty of Medicine, Vilnius University, M. K. Čiurlionio str. 21, 03101 Vilnius, Lithuania; loreta.bagdonaite@santa.lt (L.B.); sarune.naktinyte@gmail.com (S.N.)

**Keywords:** vascular endothelial growth factor, undifferentiated arthritis, disease activity markers

## Abstract

*Background and Objectives:* Early undifferentiated arthritis (UA) is a group of inflammatory joint diseases that are not classified under any specific rheumatic or connective tissue disorder and might evolve into chronic inflammatory arthritis or may be a self-limiting condition. Early recognition and treatment are crucial for the future course of the disease. Vascular endothelial growth factor (VEGF) is an angiogenic regulator that induces the growth of new capillary blood vessels, which are important in joint invasion and destruction during the progression of chronic inflammatory arthritis. The aim of this study was to assess VEGF levels associated with sociodemographic, clinical, laboratory, and ultrasound findings in the early UA patient cohort as well as to evaluate VEGF as a potential prognostic marker for arthritis outcomes. *Materials and Methods:* Seventy-six patients with inflammatory arthritis in at least one joint, with a duration of arthritis <12 months at the study entry that did not meet any rheumatic disease classification criteria, were enrolled after informed consent was obtained. Patient’s sociodemographic, laboratory data, and clinical disease characteristics were recorded, VEGF levels were measured, and ultrasound (US) of tender and swollen joints was performed. *Results:* VEGF levels had positive correlation with conventional rheumatic disease activity and diagnostic markers: erythrocyte sedimentation rate (ESR), C–reactive protein (CRP), and rheumatoid factor (RF) (*p* < 0.05). RF-positive patients had higher VEGF values (*p* = 0.024). A statistically higher number of patients whose VEGF levels were below the median value presented with active infection (*p* = 0.046). In patients with a higher number of swollen joints, and a higher score of synovitis and power doppler (PD) seen on US, VEGF levels were statistically significantly higher. Patients who after 12-month follow-up developed rheumatoid arthritis (RA) had statistically higher VEGF levels at baseline compared with those who developed spondyloarthropathies (*p* = 0.028). *Conclusions:* This study demonstrated that VEGF levels significantly represented inflammatory processes that were present in the joints (number of swollen joints, synovitis, and PD changes) of the early UA cohort.

## 1. Introduction

Undifferentiated arthritis (UA) encompasses signs and symptoms consistent with inflammatory arthritis that do not meet classification criteria for any specific rheumatic disease [1,2]. The frequency of UA ranges from 23% to 81% in early arthritis cohorts with most of them reporting a rate of 30% [3]; therefore, UA is a common diagnosis in daily rheumatology practice [4,5,6]. UA can be an early manifestation of defined arthritis, such as rheumatoid arthritis (RA), psoriatic arthritis (PsA), axial or peripheral spondylarthritis (SpA), systemic lupus erythematosus (SLE), an overlap between two or more rheumatic diseases, or a self-limited syndrome of unknown cause that resolves on its own [3,7,8].

While up to 20–60% of UA cases can resolve spontaneously, about one-third will progress into RA and the rest into other chronic rheumatic conditions [1,3]. There are baseline markers that mostly correlate with UA progression into chronic inflammatory arthritis, such as high disease activity score, presence of high levels of serum rheumatoid factor (RF) [9], and anti-cyclic citrullinated peptide (anti-CCP) [10,11]. To calculate possible UA evolvement in RA, the Laden prediction rule has been developed [12]; however, it has been demonstrated that neither anti-CCP nor the prediction rule are able to subsequently identify the individual RA patients who do not fulfill the American College of Rheumatology/European League Against Rheumatism (ACR/EULAR) 2010 rheumatoid arthritis classification criteria [13]. Sensitivity of Classification Criteria of Psoriatic Arthritis (CASPAR) [14] for early PsA has been demonstrated in a couple of studies [15,16]. It was demonstrated that CASPAR criteria have (99.1%; 95% confidence interval (95% CI) 94.9–100) sensitivity to detect early PsA, but in this study, early PsA was considered when the duration of arthritis was <2,5 years [15]. The other study enrolled patients with arthritis duration <50.9 weeks, and they found that sensitivity of CASPAR criteria went down to 77.3% [16]. As for spondylarthritis, a meta-analysis showed that The Assessment of Spondylarthritis International Society (ASAS) [17,18] criteria were 82% sensitive and 88% specific in the axial spondylarthritis group, and 87% sensitive and 63% specific in the peripheral spondylarthritis group [19]. The 2012 Systemic Lupus International Collaborating Clinics criteria (SLICC’12) [20] have demonstrated 99.5% sensitivity and 82.0% specificity [21]. Therefore, there is still a large group of patients who are in a gap and do not fulfill the classification criteria of any rheumatic disease but would benefit greatly from early treatment with disease-modifying drugs (DMARDs). Conversely, osteoarthritis (OA), which is the most prevalent arthritic disease, is often present with low grade synovitis. Even if inflammation present in OA is fundamentally different from RA [22], this leads to the fact that 0.5%–11% of UA patients can be diagnosed with OA [23]. The 2007 EULAR developed recommendations for the management of early arthritis [24], which were updated in 2016 [25]. The aim of these recommendations is early recognition of arthritis, referral, diagnosis, prognostication, and treatment [24,25], as better assessment and right monitoring of patients with early arthritis serves to better adapt therapeutic strategies [24,26]. Despite all existing diagnostic markers and early arthritis management recommendations, we are still in great need of developing new diagnostic markers for earlier and better UA outcomes prediction. 

Vascular endothelial growth factor (VEGF) is a potent angiogenic regulator involved in blood vessel formation, mitogenesis, epithelial and endothelial cell activation, and proliferation, and it has the capacity to induce both physiological and pathological angiogenesis [27,28,29,30,31]. There are six isoforms of VEGF in total, and VEGF-A predominates in the angiogenesis process [32,33]. VEGF induces proinflammatory changes in chronic inflammation such as leukocyte accumulation, collagen deposition, and blood vessel alterations [34]. Increased vascularity is one of the key features of RA synovitis [29,35,36] since active angiogenesis leads to joint invasion, destruction, and pain in the pathogenesis of chronic arthritis [36,37]. It has been demonstrated that VEGF is a key factor in the formation and maintenance of pannus [35,38] and is abundantly expressed in the synovial fluid and serum of RA patients [38,39,40]. A meta-analysis that investigated the association between VEGF and autoimmune diseases demonstrated that VEGF levels were statistically higher in RA, PsA, SpA, SLE, systemic sclerosis (SSc), Kawasaki disease, and Behcet’s disease patients, compared with healthy controls [41]. It was also demonstrated that in active SLE, VEGF was statistically higher than in inactive SLE, as well as in SLE with renal involvement compared with patients without renal involvement [41]. Meta-analyses showed significantly positive correlation between VEGF levels and disease activity in RA and with C reactive protein (CRP) levels [40,41]. Moreover, VEGF receptors are expressed in the synovium of inflamed joints in RA patients [40]. The PsA study also demonstrated that patients with active PsA had statistically higher VEGF levels than patients with inactive PsA and healthy controls. VEGF levels also correlated with erythrocyte sedimentation rate (ESR), Health Assessment Questionnaire (HAQ), psoriasis area and activity index (PASI) and visual analogue scale (VAS) [42]. VEGF has also been described as a prognostic biomarker for axial SpA, as elevated VEGF levels were associated with higher disease activity (Bath Ankylosing Spondylitis Disease Activity Index (BASDAI), Bath Ankylosing Spondylitis Metrology Index (BASMI)) and increased risk for radiographic progression [43]. The other study found that VEGF levels were elevated in different forms of arthritis (RA, PsA, reactive arthritis), but no statistical difference was found between groups [44].

The aim of this study was to assess VEGF level profile in the early UA patient cohort during first visit to a rheumatologist office, assess the association with sociodemographic, clinical, laboratory and ultrasound findings, and evaluate VEGF potential as a prognostic marker for arthritis outcome.

## 2. Materials and Methods

### 2.1. Study Design

#### 2.1.1. Study Population

This was a prospective cohort study. Patients were recruited for this study when admitted to a rheumatologist consultation at Vilnius University Hospital, Santaros Klinikos, Rheumatology center to clarify the diagnosis of inflammatory arthritis (from July 2016 to December 2017). The study was approved by the Vilnius Regional Biomedical Research Ethics Committee (approval no. 158200-15-800-310). All patients signed informed consent before they were recruited for the study.

Inclusion criteria: adult patients (>18 years old) presenting with inflammatory arthritis at least in one joint, as assessed by treating rheumatologist; patient-reported duration of arthritis was less than 12 months. Exclusion criteria: at the study entry, patients were not diagnosed with any inflammatory rheumatic disease and did not meet any of rheumatic disease classification criteria: ACR/EULAR 2010 RA classification criteria for RA [13], CASPAR for PsA [14], ASAS for axial and peripheral spondylarthritis [18], and SLICC’12 criteria for systemic lupus erythematosus [20], or another inflammatory rheumatic disease, joints swelling was deemed due to septic arthritis, arthritis due to trauma or mechanical lesions, crystal arthropathies, osteoarthritis, or paraneoplastic arthritis. [45]

At study baseline visit, each patient’s sociodemographic data (age, gender, smoking history, education level, presence of rheumatic disease in blood relatives, weight, height, body mass index (BMI)), and clinical disease characteristics (presence of comorbidities and active infection, patient-reported duration of arthralgia and joint swelling; 68 tender joint count (68 TJC) and 66 swollen joint count (66 SJC); patient’s global assessment of disease activity on 100 mm visual analog scale (patient’s global VAS), physician’s global assessment of disease activity on 100 mm visual analog scale (physician’s global VAS), patient’s joint pain assessment on 100 mm visual analog scale (patient’s joint pain VAS); disease activity score 28 (DAS 28) calculated based on assessment of 28 tender and swollen joints and ESR [46,47], Modified Health Assessment Questionnaire (MHAQ) score [48]) were recorded.

#### 2.1.2. Laboratory Tests Analysis

Blood samples were collected at the baseline visit for the detection of ESR, CRP, RF, and VEGF levels. Patients’ venous blood was drawn on an empty stomach, avoiding lipemia. Serum samples were tested in this assay for the quantitative determination of CRP, RF, and human VEGF-A. Anti-CCP, anti-nuclear antibodies (ANA), human leukocyte antigen B27 (HLA B27) analysis was performed at the discretion of the treating rheumatologist as clinically indicated. The normal ranges of laboratory tests and units are presented in Table 1.

Before the investigation, serum samples were stored frozen at −20 °C. Prior to assay, the frozen samples were brought to room temperature slowly and mixed gently. Enzyme-linked immunosorbent assay (ELISA) was performed to measure VEGF-A levels in sera, following the manufacturer’s instructions. ELISA kit for human VEGF-A was from IBL International, Germany (catalog no. BE55101). Samples were analyzed in duplicate.

#### 2.1.3. Ultrasound Examination

At baseline, ultrasound of all tender and swollen joints was performed for all patients (General Electric LOGIQ E Portable Ultrasound). Synovitis, power Doppler (PD), and erosion findings were scored using a scale of 0 to 3. Scores from each joint were added up to calculate synovitis, PD, and erosions (Table 2) [49,50].

#### 2.1.4. Outcome Measurements and VEGF Profile Evaluation

Patients were followed for 12 months. UA outcome was either diagnosis confirmed based on rheumatic diseases classification criteria [14,17,18,20,45] or confirmed that arthritis resolved completely. Diagnoses were confirmed based on classification or diagnostic criteria: (1) RA established based on ACR/EULAR 2010 RA classification criteria [13]; (2) SpA (reactive arthritis established in patients present with monoarthritis/oligoarthritis and confirmed urethritis, Lyme disease, diarrhea, etc. [51,52,53]), axial or peripheral spondylarthritis established based on ASAS criteria [17,18], PsA established based on CASPAR classification criteria [14]; (3) other autoimmune inflammatory diseases (SLE established based on SLICC’12 classification criteria [20], undifferentiated connective tissue disease (UCDT) established for patients who demonstrated clinical and serological characteristics of a connective tissue disease but did not fulfill specific classification criteria [54,55], IgG 4-related disease (IgG4-RD) established based on comprehensive diagnostic criteria for IgG4-RD [56]); (4) patients whose arthritis resolved completely—remission seen by 6 months follow-up (no swollen joints) without need for steroids or DMARDs. The final diagnosis of patients who did not attend a follow-up visit after 12 months was verified by reviewing the medical electronic records.

VEGF importance in UA diagnostic was analyzed. Patients were divided into two groups based on VEGF median value; differences between sociodemographic, clinical disease characteristics, laboratory and ultrasound findings were evaluated. As there are no established VEGF cutoff values in clinical practice to determine the activity of arthritis, and VEGF level distribution was nonparametric, we chose to use the median as the cutoff value. Correlation of VEGF value with all analyzed parameters was evaluated. VEGF value in patient groups were divided by: age according to median value and according to the World Health Organization (WHO), established age group classification (18–47, 48–63 and ≥64 years of age) gender, smokers and nonsmokers, education level (divided by median), BMI (normal weight BMI < 25 kg/m^2^/overweight BMI ≥ 25 kg/m^2^ [57]), family medical history, other clinical disease characteristics, and ultrasound data (quantitative data grouped based on mean or median), ESR and CRP (normal/elevated range), RF, anti-CCP, ANA (positive or negative), HLA B27 antigen (expressed or not expressed).

VEGF value distribution among different UA outcomes was analyzed: established RA vs. other study population (spondyloarthropathies, patients whose arthritis resolved, and other autoimmune inflammatory condition), patients whose arthritis resolved vs. other study population (spondyloarthropathies, RA, and other autoimmune inflammatory condition), etc. The prognostic value of the VEGF test for the prediction of early inflammatory arthritis outcomes was evaluated.

### 2.2. Statistical Analysis

Statistical analysis was conducted using IBM SPSS 25 software (SPSS Chicago, IL, USA) and the data are represented as mean ± standard deviation (SD), median (minimum and maximum) values, or percentages. Data normality was checked using Shapiro–Wilk test. Since the variables did now show normal distribution, non-parametric tests were more suitable. Correlation was tested using Spearman’s method to determine the relationship between VEGF levels and sociodemographic, laboratory, clinical, and ultrasound variables. For quantitative data, Mann–Whitney U test and Kruskal–Wallis, and for qualitative, Chi-Square tests, were used. Association strength between risk for UA to progress into chronic inflammatory arthritis and VEGF levels was assessed by computing odds ratio (OR) with 95% confidence intervals (95%CI). Statistical significance level was set at 0.05.

## 3. Results

### 3.1. Sociodemographic, Clinical, Laboratory and Ultrasound Parameters in Undifferentiated Arthritis Patient Cohort and Their Association with VEGF Levels

Seventy-six patients, mean age 43 ± 15.81 years, were enrolled in the study; 51 (6.71%) of them were females; thirteen (17.1%) were daily smokers; mean education-13.49 ± 2.05 years; BMI of patients was 24.02 ± 3.18 kg/m^2^; 27 (35.5%) reported a history of rheumatic diseases in blood relatives (RA, PsA, SpA, SLE and etc.). At the study entry mean duration of joint pain was 6.9 ± 5.2 months, joint swelling was 5.42 ± 3.25 months. All sociodemographic, clinical, laboratory and ultrasound data are presented in Table 3. At the time of enrollment, median VEGF level was 365.27 (minimum value 25.75; maximum value 3438.23) pg/mL. Between patient groups divided by VEGF median value (Table 3), no statistical difference was found between sociodemographic data (age, gender, daily smokers, education in years, BMI, presence of rheumatic diseases in family), as well as clinical variables such as presence of comorbidities, duration of joint pain and swelling in months, duration of morning stiffness, patient’s joint pain VAS, patient’s global VAS, physician’s global VAS, MHAQ score, or DAS 28 score. Number of patients with active infection was statistically higher in the patient group with VEGF levels lower than the median value (*p* = 0.046). In the patient group with VEGF values higher than the median, there was a statistically significantly higher number of swollen joints than in the group with lower than median value: 66 SJC (4.0 ± 2.7 and 2.5 ± 1.37, respectively; *p* = 0.019), and 28 SJC (3.37 ± 1.85 and 2.18 ± 1.16, respectively; *p* = 0.016), and there was no statistical differences between 68 and 28 TJC. In the patient group where VEGF levels were above the median, values were significantly more in patients with elevated CRP, positive RF and anti-CCP values (*p* = 0.039, *p* = 0.014, and *p* = 0.041, respectively). No statistical difference was found between patient number in elevated ESR values, expressed HLA B27, and positive ANA test patient groups. Between patient groups divided by the VEGF median value, the grade of synovitis and grade of erosions seen in the US were statistically higher in the group with higher than the median VEGF values (*p* = 0.049 and 0.018, respectively) (Table 3).

Correlation of VEGF concentration with patient age, education level, and BMI has not been confirmed. In the clinical data analysis, weak positive correlations between VEGF levels and 66 SJC (r = 0.428, *p* = 0.006) and 28 SJC (r = 0.375, *p* = 0.001) were confirmed. Conversely, correlation between 68 TJC and 28 TJC was not confirmed as well as between VEGF levels and duration of joint pain, joint swelling, morning stiffness, patient’s joint pain and global VAS, and physician’s global VAS, MHAQ score, and DAS 28. VEGF value had a weak positive correlation with ESR (r = 0.256, *p* = 0.029), CRP (r = 0.375, *p* = 0.001), and RF (r = 0.263, *p* = 0.022) values. In ultrasound analysis, VEGF levels had weak positive correlation with the grade of synovitis (r = 0.332, *p* = 0.003), PD (r = 0.370, *p* = 0.018) and grade of erosions seen on US (r = 0.256, *p* = 0.026) (Table 4).

No statistically significant difference has been detected between VEGF level distribution in patient groups divided by age, gender, BMI (divided by normal weight/overweight), education level in years (divided by median), smoking history (smokers/non-smokers), or history of rheumatic diseases in blood relatives (present/not present) (Table 5).

In the patient groups divided by mean values of affected joints, VEGF values were statistically higher in the groups above mean value of 66 SJC (788.75 and 390.34 pg/mL, respectively; *p* = 0.005) and 28 SJC (665.95 and 381.35 pg/mL, respectively; *p* = 0.004). No statistically significant differences were confirmed between VEGF value in patient groups divided by mean values in 68 TJC, 28 TJC, duration of patients reporting joint pain and swelling, patient’s joint pain and global VAS, assessor’s global VAS, MHAQ score, DAS28 and duration of morning stiffness (divided by median). No differences were found between VEGF levels in the patient groups present with comorbidities or not and presenting active infection or not (Table 6).

VEGF value was statistically significantly higher in the RF-positive (708.97 pg/mL) compared with the RF-negative (427,17 pg/mL) patient group (*p* = 0.024). VEGF value was higher but not significantly in anti-CCP-positive patient groups compared with anti-CCP negative, RF and anti-CCP positive compared with RF and anti-CCP negative (Table 7).

At baseline, 29 (38.2%) patients presented with joint erosions detected on US examination. Patients with synovitis grade above the mean value had statistically higher levels of VEGF than ones that were below (782.16 and 385.34 pg/mL, respectively; *p* = 0.007). Patient groups where PD score was above the mean value also had statistically higher VEGF values than the ones that had a lower PD score (718.21 and 398.29, respectively; *p* = 0.042) (Table 8).

### 3.2. Prognostic Value of VEGF Levels at Study Baseline in Relation with Undifferentiated Arthritis Patient Cohort Outcomes after 12 Months Follow-Up

**Figure 1:**
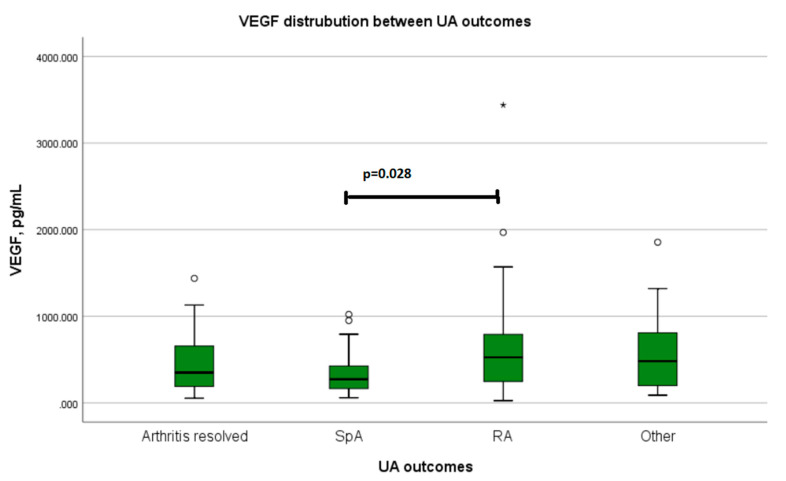
VEGF level distribution at the onset of undifferentiated arthritis between disease outcomes after 12 months of follow-up. SpA: spondyloarthropathies (reactive arthritis, axial or peripheral spondylarthritis, psoriatic arthritis); RA: rheumatoid arthritis; other: other autoimmune inflammatory diseases (systemic lupus erythematosus, undifferentiated connective tissue disease, IgG 4 related disease); UA: undifferentiated arthritis; VEGF: vascular endothelial growth factor.

After 12 months follow-up, a total of 23 patients developed RA, 23 developed spondyloarthropathy (ankylosing spondylarthritis, psoriatic arthritis, reactive arthritis), 10 developed other autoimmune inflammatory diseases (systemic lupus erythematosus, undifferentiated connective tissue disease, IgG 4 related disease), and for 20 patients, arthritis resolved completely. VEGF levels were statistically significantly lower in patients with spondyloarthropathies, compared with all other UA outcomes groups (*p* = 0.046) (Table 9) and RA separately (*p* = 0.028) (Figure 1). In the RA outcome group compared with other patients in this study, VEGF levels were higher, although not statistically significant. No significant difference was confirmed between patients whose arthritis resolved and the other study population (Table 9).

Between the patient groups divided by VEGF median value at study entry, in the group with VEGF values below the median, there was a significantly higher number of patients who were confirmed with spondyloarthropathy diagnosis after 12 months follow-up (Table 10).

Logistic regression did not show statistically significant associations between UA outcomes and VEGF levels (Table 11).

## 4. Discussion

From a clinical point of view, UA presents all spectrums of clinical and laboratory findings that do not allow for one to make any define clinical diagnosis based on current existing classification criteria as of yet [2]. From an immunological point of view, recent-onset UA represents a state of acute inflammation for which the question arises which factors are involved in its initiation and cessation, and which determine its persistence [58]. The role of VEGF in various inflammatory arthritis and connective tissue diseases pathogenesis, and its value as a potential diagnostic and disease activity marker in rheumatic diseases are highly discussed [29,40,59]. In this study, we analyzed the significance of VEGF levels in the context of differential diagnosis in an early UA patient cohort. In addition, the relationship between the main sociodemographic, clinical, laboratory and joint US parameters that are used in inflammatory rheumatic diseases diagnostics and the patient’s serum VEGF levels were assessed.

It is known that some inflammatory rheumatic joint diseases are prone to start in certain age groups [17,60,61,62], and some are more frequent between female gender, then other are more frequent between males [63,64,65,66]. We found no difference in VEGF levels between gender and age groups, and this corresponds with other author reports [67]. It is known that in the pathogenesis of RA, SpA, and PsA smoking [68,69,70,71,72], obesity [72,73,74] and lower education [74,75] are related with poor disease prognosis. Studies demonstrated that smoking is associated with oxidative stress and higher VEGF secretion [76]. It is also known that VEGF regulates adipose development [77]. In our study, we found no statistical significance between VEGF levels in smoking and non-smoking patients as well as patients within a normal weight range and obesity. No statistically significant difference was detected in VEGF levels between patients with higher and lower educations. This might be because of the relatively small study population cohort, as to confirm the impact of smoking, obesity, and education on VEGF levels requires large populations.

The present study confirmed that VEGF levels were positively associated with ESR, CRP, and RF values, and RF-positive patients had statistically significantly higher VEGF values than RF negative. Numerous studies and a meta-analysis correspond to our results [40,78,79,80,81,82]. As Anti-CCP is the most prominent RA diagnostic and prognosis marker [10,13], we confirmed that the patient group with higher VEGF values also had a statistically higher number of anti-CCP positive patients. A tendency was seen in that anti-CCP positive patients had higher VEGF values compared with negative, but they were not significant. Some authors confirmed a significant correlation between anti-CCP and VEGF [83,84], but there are also studies that detected no significant difference between VEGF levels and anti-CCP-positive and anti-CCP-negative RA patients [84]. These data suggest that VEGF may be one more serological inflammatory marker of autoimmune arthritis activity, and may possibly play a role in the differential diagnosis of early UA.

Similar to our results, other studies confirmed no significant association between VEGF levels and DAS 28, HAQ, and VAS, the duration of morning stiffness, or the number of tender joints [83,84]. In this study, we demonstrated that patients who had a higher number of swollen joints (both 66 SJC and 28 SJC) and higher scores of synovitis and PD seen on US had statistically higher VEGF levels. VEGF receptors are highly expressed in RA synovial tissues, and expression levels parallel the degree of synovial angiogenesis, which is a prerequisite for pannus formation [31,85,86,87]. Similar results have been seen in numerous US studies, where patients who presented with a higher score of synovitis and PD had higher levels of VEGF [81,88,89]. It was found that VEGF levels measured in synovial fibroblasts failed to correlate with the grey scale US, PD, or with ESR and CRP values [81]. US is widely used in daily practice as a cost-effective, noninvasive diagnostic tool for inflammatory arthritis, as well as to assess arthritis response to treatment [90]. There are attempts to develop US methodologies for early UA [91]. Most recommendations involve the assessment of 6, 12, 18, 22, 26, 32, and 38 joints [91,92,93,94,95,96,97], and are quite time-consuming, requiring up to 30 min time to perform scanning [98], which makes it hard to apply in everyday rheumatologist practice. Nevertheless, often on the onset of arthritis, mono- or oligoarthritis can be observed, and based on existing US recommendations for UA, these joints might not be involved while performing US [91,92,93,94,95,96,97]. This is why in this study, we investigated only joints that were involved (all painful and swollen joints) and confirmed that US findings had statistically significant positive correlations with VEGF levels present at the moment of first patient examination. This allowed us to make suggestions that monitoring VEGF levels, as well as other disease activity markers, may help us to better evaluate UA synovitis activity and response to treatment, and might serve as a red flag indicating the presence of active synovitis and the threat of erosive arthritis.

It has been confirmed that VEGF importance in the etiopathogenesis of many diseases and elevated VEGF levels have been detected in multiple sclerosis, malignancies, etc. [30,99]. In our study, no statistical difference between patients who presented with comorbidities and those who did not was confirmed. This allowed us to suggest that in this study, VEGF levels were determined by the activity of arthritis and not by the presence of concomitant noninfectious diseases [100].

The importance of infections in the etiopathogenesis of arthritis is known. Bacteria, viruses, fungi, and parasites can all cause arthritis of either acute or chronic nature [101]. In other cases, suspicion of the possible presence of infection postpones arthritis treatment with disease-modifying antirheumatic drugs (DMARDs), as most of them are immunosuppressive agents [102]. There are numerous studies that have found VEGF association with viral infections, such as Coronavirus disease 2019 (COVID-19) [103,104,105,106] and Epstein–Barr virus (EBV) [107]. Conversely, a malaria study demonstrated that patients with severe disease had significantly lower levels of VEGF compared with patients who had a mild form of the disease [108], as well as a tuberculosis (TB) study that found that patients with active TB had statistically lower VEGF levels than patients with latent TB, and patients with acute bronchitis had lower VEGF levels than the healthy control group [109,110]. In this study, we found that patients who presented with active infection (bacterial, such as *Chlamydia trachomatis, Yersinia enterocolitica,* Lyme disease, acute tonsilitis, etc.) had statistically lower VEGF levels than ones with no infection. Conversely, we also confirmed that patients with higher VEGF levels presented with higher CRP. These results allowed us to propose that in the early stages of inflammatory arthritis, VEGF levels might allow us to differentiate patients whose CRP is elevated due to active infection and those to whom it might be elevated because of active arthritis, and it would help to introduce treatment with DMARDs earlier. VEGF concentration was found to be significantly different in various rheumatic diseases, including RA, SLE, antiphospholipid syndrome (AFS), and mixed connective tissue diseases [111,112,113,114]. SLE studies confirmed VEGF association with disease activity [112,113,114]. Other studies found statistically lower VEGF levels in healthy controls compared with SLE [115], as well as AS patients [116,117,118]. A meta-analysis has demonstrated that VEGF levels may be a marker of inflammatory activity in RA and may play an important role in the inflammatory process, and higher VEGF levels were strongly correlated with the presence of RA [40]. RA compared with SLE and AFS had statistically higher expression levels of VEGF [111]. VEGF had also been described to be a prognostic biomarker for axial SpA, with elevated VEGF levels being associated with an increased risk for radiographic progression of the disease [43]. In our study, the main endpoint was the patients’ diagnosis after 12-month follow-up. The study revealed that patients who after 12-month follow-up developed spondyloarthropathy had statistically lower VEGF levels compared with patients who developed RA, as well as with all the other study populations (those who developed other autoimmune inflammatory conditions or whose arthritis resolved completely). The patient group with lower VEGF levels had a significantly higher number of patients diagnosed with spondyloarthropathies. We can suggest that these results correspond to differences between RA and spondyloarthropathies classification criteria [13,17,18], as patients with spondyloarthropathies are RF anti-CCP negative and usually present with mono- or oligoarthritis, and in this study, patients who presented with these clinical and laboratory findings had significantly lower levels of VEGF.

In this early UA cohort study, we confirmed that higher VEGF values were statistically significantly associated with higher levels of CRP, ESR, number of swollen joints, and higher scores of synovitis and PD seen on US. In the patient group where VEGF values were above the median, a statistically higher number of patients with both positive RF and anti–CCP was detected. ESR, CRP, RF, anti–CCP, and number of swollen joints are included in RA classification criteria [106] and higher ESR, CRP, and positive RF with anti–CCP are known as poor prognosis markers in RA [119]. This study also demonstrated that VEGF levels significantly represented inflammatory processes that were present in the joints (number of swollen joints, synovitis, and PD changes). We also found that VEGF levels detected at the onset of the inflammatory joint disease were statistically different between the two main inflammatory joint diseases, which was higher in the patient group that was diagnosed with RA after 12-month follow-up compared with patients that were diagnosed with spondyloarthropathies. 

## 5. Conclusions

To conclude, in our study, we confirmed that elevated VEGF levels were statistically associated with poor RA prognosis markers (positive RF and anti–CCP, ESR and CRP values, swollen joints count) and changes seen on US (synovitis, PD score, erosions grade). The fact that patients with active bacterial infection had lower VEGF levels than ones with no infection might also suggest that VEGF might be helpful in making decisions about inflammation etiology and which treatment should be introduced. All these results allowed us to suggest that VEGF might also be introduced as a promising diagnostic marker in early inflammatory arthritis differentiation, which might increase the sensitivity and specificity for RA and other inflammatory diseases regarding diagnostic or classification criteria, although further investigation is needed.

## Figures and Tables

**Table 1:** Description of laboratory tests.

Analyte	Cutoff	Units	Method Used	Supplier of the Reagents Kits
ESR	20	mm/h	Westergren method	Automated System Test I, Alifax, Polverara, Italy
CRP	5	mg/L	Nephelometry	BN II System, Siemens Healthcare GmbM, Erlangen, Germany
RF	30	kU/L	Nephelometry	
Anti-CCP	5	U/ml	ELISA	EUROIMMUN Medizinische Labordiagnostika AG, Lübeck, Germany
ANA	1:40	titer	Indirect immunofluorescence	
HLA B 27	presentation	expression	Flow cytometry	Becton Dickinson, Franklin Lakes, NJ, USA
VEGF	43	pg/ml	ELISA	IBL International, Hamburg, Germany

ESR, erythrocyte sedimentation rate; CRP, C-reactive protein; RF, rheumatoid factor; Anti-CCP, anticitrullinated protein antibodies; ANA, antinuclear antibodies; HLA B27, human leukocyte antigen B27; VEGF, vascular endothelial growth factor; ELISA, enzyme-linked immunosorbent assay.

**Table 2:** Ultrasound imaging: synovial, Power Doppler and bone changes.

Pathologic Change	Sonographic Scoring System
Synovitis and grey scale (GS)	None = 0 Mild = 1 Moderate = 2 Severe = 3
Power Doppler (PD)	No flow in the synovium = 0 Single vessel signals = 1 Confluent vessel signals in less than half of the area of the synovium = 2 Vessel signals in more than half of the area of the synovium = 3
Erosions	None = 0 Surface irregularity where no defect detected in 2 perpendicular planes = 1 Surface defect seen in 2 perpendicular planes = 2 Defect forming severe bone destruction = 3

**Table 3:** Sociodemographic, clinical, laboratory and ultrasound parameters and their distribution based on VEGF median levels in the undifferentiated arthritis patient cohort.

Variables	Variable Results *	VEGF < 365.27 pg/mL, *n* = 38 *	VEGF ≥ 365.27 pg/mL, *n* = 38 *	*p* Value **
**Sociodemographic Variables**				
Age, years	43.03 ± 15.81	40.08 ± 14.39	43.97 ± 17.34	0.639
Female (%)	51 (67.1)	27 (71.1)	24 (63.3)	0.464
Male (%)	25 (32.9)	11 (28.9)	14 (36.8)	
Daily smokers (%)	13 (17.1)	8 (21.1)	5 (13.2)	0.361
Education, years	13.49 ± 2.05	13.74 ± 2.23	13.24 ± 1.85	0.406
BMI, kg/m^2^	24.02 ± 3.18	24.15 ± 3.23	23.89 ± 3.17	0.747
BMI, kg/m^2^ (≥25 kg/m^2^, overweight) (%)	32 (42.1)	17 (44.7)	15 (39.5)	0.642
Presence of rheumatic diseases in family (%)	27 (35.5)	16 (42.1)	11 (28.9)	0.231
**Clinical data variables**				
Patient’s joint pain VAS, mm	45.56 ± 16.74	45.82 ± 15.35	45.29 ± 18.23	0.688
Patient’s global VAS, mm	48.26 ± 16.76	47.05 ± 15.68	49.47 ± 17.89	0.770
Physician’s global VAS, mm	46.17 ± 14.03	45.23 ± 12.96	46.82 ± 15.18	0.828
Duration of joint swelling, months	5.42 ± 3.25	5.50 ± 3.19	5.34 ± 3.35	0.778
Duration of joint pain, months	6.9 ± 5.2	6.61 ± 4.36	7.24 ± 5.96	0.946
Duration of morning stiffness, min	30 (0–300)	61.05 (0–300)	71.84 (0–300)	0.697
Presence of comorbidities (%)	51 (67.1)	23 (65.5)	28 (73.7)	0.222
Presence of active infection ^1^ (%)	26 (34.2)	16 (41.2)	10 (26.3)	**0.046**
66 SJC	3.25 ± 2.26	2.5 ± 1.37	4.0 ± 2.7	**0.019**
28 SJC	2.78 ± 1.65	2.18 ± 1.16	3.37 ± 1.85	**0.016**
68 TJC	7.47 ± 5.19	7.13 ± 6.36	7.82 ± 5.25	0.484
28 TJC	5.47 ± 6.35	5.26 ± 4.07	5.68 ± 3.24	0.286
DAS 28 (ESR)	4.5 ±1.07	4.35 ± 1.09	4.65 ± 1.05	0.270
MHAQ score	0.6 ± 0.42	0.64 ± 0.43	0.56 ± 0.40	0.421
**Laboratory data variables**				
VEGF, pg/ml	365.27 (25.75–3438.23)	-	-	-
ESR, mm/h	26 (2–144)	16 (2–110)	37 (2–114)	0.130
ESR, >20 mm/h (%)	41 (56.2)	16 (45.7)	25 (65.8)	0.084
CRP, mg/L	0.7 (0.16–144.30)	12.57 (0.16–85.70)	25.80 (0.39–144.30)	0.127
CRP, >5 mg/L (%)	37 (48.7)	14 (36.8)	23 (60.5)	**0.039**
RF, kU/L	20 (9.59–814.2)	-	-	-
RF positive, >30 kU/L (%)	24 (31.6)	7 (18.4)	17 (44.7)	**0.014**
Anti-CCP, U/ml	2 (2–300)	-	-	-
Anti-CCP ^3^ positive, ≥5 U/mL (%)	23 (31.9)	8 (22.4)	15 (41.7)	**0.041**
RF and anti-CCP ^3^ positive (%)	22 (30.6)	7 (19.4)	15 (41.7)	**0.041**
HLA B27 ^4^ positive (%)	17 (28.8)	10 (30.3)	7 (26.9)	0.776
ANA ^5^ positive, titer >1:40 (%)	14 (41.2)	7 (38.9)	7 (43.8)	0.774
**Ultrasound findings**				
Synovitis score	6.46 ± 5.29, 5 (3–30)	5.24 ± 3.73	7.68 ± 6,31	**0.049**
Power Doppler score	3.17 ± 3.12, 2 (0–14)	2 (0–14)	3 (3–12)	0.329
Erosions (grade) median	0 (0–10); 1.33 ± 2.21	0 (0–8)	0 (0–10)	**0.018**
Presence of erosions (%)	29 (38.2)	11 (28.9)	18 (47.4)	0.098

* Continuous data are presented and median (minimum and maximum) values or mean ± standard deviation counts as numbers and valid percentages. ** *p* value was calculated between groups divided by VEGF median value. BMI, body mass index; VAS, visual analogue scale; SJC, swollen joints count; TDJ, tender joints count; DAS 28 (ESR), disease activity score 28 using on erythrocyte sedimentation rate; MHAQ, Modified Health Assessment Questionnaire; VEGF, vascular endothelial growth factor; ESR, erythrocyte sedimentation rate; CRP, C-reactive protein; RF, rheumatoid factor; Anti-CCP, anticitrullinated protein antibodies; HLA B27, human leukocyte antigen B27; ANA, antinuclear antibodies; PD, Power Doppler; *p* significant if <0.05; values with statistical significance are bolded. Total tested: ^1^ *n* = 52, ^3^ *n* = 72, ^4^ *n* = 59, ^5^ *n* = 34.

**Table 4:** VEGF level correlation between early undifferentiated arthritis sociodemographic, clinical, laboratory and ultrasound data.

Variables	Correlation Coefficient, r	Statistical Significance, *p*
**Sociodemographic variables**		
Age, years	0.075	0.521
Education, years	−0.108	0.355
BMI, kg/m^2^	0.002	0.986
**Clinical data variables**		
Patient’s joint pain VAS, mm	0.017	0.884
Patient’s global VAS, mm	0.017	0.542
Physician’s global VAS, mm	0.063	0.588
Duration of joint swelling, months	0.085	0.466
Duration of joint pain, months	0.007	0.949
Duration of morning stiffness, min	0.174	0.133
66 SJC	**0.428**	**0.006**
28 SJC	**0.375**	**0.001**
68 TJC	0.148	0.203
28 TJC	0.138	0.235
DAS 28 (ESR)	0.148	0.209
MHAQ score	0.044	0.709
**Laboratory data variables**		
ESR, mm/h	**0.256**	**0.029**
CRP, mg/L	**0.375**	**0.001**
RF, kU/L	**0.263**	**0.022**
Anti-CCP, U/mL	0.171	0.151
**Ultrasound findings**		
Synovitis score	**0.332**	**0.003**
PD score	**0.370**	**0.018**
Erosion’s grade,	**0.256**	**0.026**

BMI, body mass index; VAS, visual analogue scale; SJC, swollen joints count; TDJ, tender joints count; MHAQ, Modified Health Assessment Questionnaire; DAS 28 (ESR), disease activity score 28 using on erythrocyte sedimentation rate; ESR, erythrocyte sedimentation rate; CRP, C-reactive protein; RF, rheumatoid factor; Anti-CCP, anticitrullinated protein antibodies; HLA B27, human leukocyte antigen B27; ANA, antinuclear antibodies; VEGF, vascular endothelial growth factor; PD, Power Doppler, *p* significant if <0.05; values with statistical significance are bolded.

**Table 5:** VEGF level distribution between early undifferentiated arthritis sociodemographic parameters data.

Sociodemographic Variable	*n* (%)	VEGF (pg/mL) *	Statistical Significance between Groups, *p*
Age ^1^: <43 years	30 (39.5)	533.24 (25.75–3438.23)	0.302
≥43 years	46 (60.5)	493.15 (58.47–1967.50)	
Age ^2^: (18–47) years	44 (57.9)	338.04 (25.75–3438.23)	0.758
(48–63) years	20 (26.3)	409.47 (154.73–1130.02)	
>64 years	12 (15.8)	457.20 (58.47–1967.50)	
Females	51 (67.1)	541.84 (25.75–3438.23)	0.829
Males	25 (32.9)	463.77 (53.97–1437.35)	
Daily smokers	13 (17.1)	492.60 (115.88–1570.04)	0.735
Non-smokers	63 (82.9)	521.01 (25.75–3438.23)	
Education: <13.49 years	30 (39.5)	469.06 (53.97–1570.04)	0.832
≥13.49 years	46 (60.5)	546.87 (25.75–3438.23)	
BMI ^3^: normal weight	44 (57.9)	524.82 (25.75–3438.23)	0.979
overweight	32 (42.1)	504.24 (53.97–1967.500)	
Presence of rheumatic diseases in family	27 (35.5)	496.90 (58.47–3438.23)	0.525
No history of rheumatic diseases in family	49 (64.5)	526.77 (25.75–1967.50)	

* Continuous data are presented in median (minimum and maximum). *n*, number of patients; BMI, body mass index; VEGF, vascular endothelial grow factor; *p* significant if <0.05. ^1^ above or below mean value, ^2^ age groups based on WHO (World health Organization), ^3^ normal weight is BMI < 25 (kg/m^2^), overweight is BMI ≥ 25 kg/m^2^.

**Table 6:** VEGF level distribution between early undifferentiated arthritis patient cohort clinical variable data results.

Clinical Variable	*n* (%)	VEGF (pg/mL) *	Statistical Significance between Groups, *p*
Duration of joint pain ^1^: <6.9 months ≥6.9 months	39 (51.3)	527.73 (25,75–3438.23)	1.000
	37 (48.7)	503.96 (53.94–1854.91)	
Duration of joint swelling ^1^: <52.42 months ≥5.42 months	38 (50)	460.46 (25.75–1854.91)	0.938
	38 (50)	571.85 (53.97–3438.23)	
Duration of morning stiffness ^2^: < 30 min ≥30 min	22 (28.9)	432.19 (87.81–1570.04)	0.610
	54 (71.1)	550.37 (25.75–3438.23)	
Patient’s joint pain VAS ^1^: ≤45 mm >45 mm	46 (60.5)	547.81 (25.75–3438.23)	0.928
	30 (39.5)	467.50 (53.97–1967.50)	
Patient’s global VAS ^1^: ≤48 mm >48 mm	51 (67.1)	526.97 (25.75–3438.23)	0.627
	25 (32.9)	494.09 (53.97–1967.50)	
Physician’s global VAS ^1^: ≤46 mm >46 mm	49 (64.5)	527.94 (25.75–3438.23)	0.649
	27 (35.5)	494.76 (53.97–1967.50)	
MHAQ score ^1^: <0.6 ≥0.6	37 (48.7)	533.42 (87.82–3438.23)	0.897
	39 (51.3)	499.78 (25.75–1967.50)	
Presence of comorbidities Absence of comorbidities	51 (67.1)	504.53 (58.47–1967.50)	0.296
	25 (32.9)	539.88 (25.75–3438.23)	
Presence of active infection ^3^ Absence of active infection	26 (34.2)	355.25 (58.47–1130.02)	0.487
	26 (34.2)	496.73 (53.97–3438.23)	
66 SJC ^1^: ≤3.25 >3.25	52 (68.4)	390.34 (25.75–1437.35)	**0.005**
	24 (31.6)	788.75 (58.47–3438.23)	
28 SJC ^1^: ≤2.78 >2.78	40 (52.6)	381.35 (25.75–1967.50)	**0.004**
	36 (47.4)	665.95 (58.47–3438.23)	
68 TJC ^1^: ≤7.47 >7.47	47 (61.8)	404.66 (53.97–1437.35)	0.067
	29 (38.2)	696.86 (25.7–3438.23)	
28 TJC ^1^: ≤5.47 >5.47	43 (56.6)	409.71 (53.97–1437.35)	0.176
	33 (43.4)	654.86 (25.75–3438.23)	
DAS 28 ^1^, score: <4.5 ≥4.5	33 (45.8)	504.12 (53.97–1967.50)	0.769
	39 (54.2)	557.05 (25.75–3438.23)	

* Continuous data are presented in median (minimum and maximum) values. *n*, number of patients; VAS, visual analogue scale; MHAQ, Modified Health Assessment Questionnaire; SJC, swollen joints count; TDJ, tender joints count; DAS 28 (ESR), disease activity score 28 calculated based on assessment of 28 joint and erythrocyte sedimentation rate (ESR); VEGF, vascular endothelial growth factor; *p* significant if <0.05; values with statistical significance are bolded. ^1^ variable groups are split according to variables means values; ^2^ variable groups are split according to variable median values. Total tested: ^3^ *n* = 52.

**Table 7:** VEGF level distribution between undifferentiated arthritis patient cohort laboratory test results.

Laboratory Test	*n* (%)	VEGF (pg/mL) *	Statistical Significance between Groups, *p*
ESR ^1^, mm/h ≤ 20 >20	32 (43.8)	413.01 (53.97–1570.04)	0.186
	41 (56.2)	618.65 (25.75–3438.23)	
CRP ^1^, mg/L ≤ 5.0 >5.0	39 (51.3)	375.85 (53.97–1570.04)	0.081
	37 (48.7)	664.03 (25.75–3438.23)	
RF, positive negative	24 (31.6)	708.97 (25.75–3438.23)	**0.024**
	52 (68.4)	427.17 (53.97–1854.91)	
Anti-CCP ^2^, positive	23 (31.9)	695.72 (25.75–3438.23)	0.093
negative	49 (68.1)	446.63 (53.97–1854.91)	
RF and anti-CCP, positive negative	22 (30.6)	714.46 (25.75–3438.23)	0.073
	50 (69.4)	443.36 (53.97–1854.91)	
HLAB27 ^3^, expressed	17 (28.8)	318.83 (68.67–661.84)	0.284
not expressed	42 (71.2)	520.41 (25.75–1967.50)	
ANA ^4^, positive	14 (41.2)	488.19 (87.82–1854.91)	0.649
negative	20 (58.8)	615.98 (140.64–3438.23)	

* Continuous data are presented in median (minimum and maximum). *n*, number of patients; RF, rheumatoid factor; Anti-CCP, anticitrullinated protein antibodies; ANA, antinuclear antibodies; HLA B27, human leukocyte antigen B27; ESR, erythrocyte sedimentation rate; CRP, C-reactive protein; VEGF, vascular endothelial grow factor; *p* significant if <0.05; values with statistical significance are bolded; ^1^ variable groups are split according to normal values or elevated range. Total tested: ^2^ *n* = 72, ^3^ *n* = 59, ^4^ *n* = 34.

**Table 8:** VEGF level distribution between undifferentiated arthritis patient cohort in ultrasound results.

Ultrasound Variable	*n* (%)	VEGF (pg/mL) *	Statistical Significance between Groups, *p*
Synovitis score ^1^: ≤6.46	51 (67.1)	385.34 (25.75–1437.35)	**0.007**
>6.46	25 (32.9)	782.16 (58.47–3438.23)	
Power Doppler score ^1^: <3.17	48 (63.2)	398.29 (25.75–1437.35)	**0.042**
≥3.17	28 (36.8)	718.21 (58.47–3438.23)	
Presence of erosions Absence of erosions	29 (38.2)	639.06 (58.47–3438.23)	0.074
	47 (61.8)	440.32 (25.75–1968.50)	

* Continuous data are presented in median (minimum and maximum). *n*, number of patients; VEGF, vascular endothelial growth factor; *p* significant if <0.05; values with statistical significance are bolded. ^1^ variables groups are split according to mean values.

**Table 9:** VEGF level distribution between undifferentiated arthritis patient cohort outcomes in 12-month follow-up.

Diagnosis	Patients with Confirmed Diagnosis (*n*)	*p* Value	VEGF (pg/mL) in Confirmed Diagnosis Patient Group *	VEGF (pg/mL) in Other Study Population *	*p* Value
Rheumatoid arthritis	23	0.108	525.65 (25.75–3438.23)	297.43 (53.97–1854.91)	0.134
Spondyloarthropathies ^1^	23		272.3 (58.47–1020.74)	412.77 (25.75–3438.23)	**0.046**
Arthritis resolved	20		350.03 (53.97–1437.35)	372.04 (25.75–3438,23)	0.794
Other autoimmune inflammatory disease ^2^	10		481.59 (87.82–1854.91)	353.27 (25.75–3438.23)	0.734

* Continuous data are presented in median (minimum and maximum). *n*, number of patients; ^1^ spondyloarthropathies: reactive arthritis, axial or peripheral spondylarthritis, psoriatic arthritis; ^2^ other autoimmune inflammatory diseases: systemic lupus erythematosus, undifferentiated connective tissue disease, IgG 4 related disease; VEGF; vascular endothelial growth factor. Continuous data are presented and median (minimum and maximum) values; *p* significant if <0.05; values with statistical significance are bolded.

**Table 10:** Association between undifferentiated arthritis study cohort outcomes based on VEGF median levels in undifferentiated arthritis patient cohort.

Diagnosis	VEGF< 365.27 pg/mL, *n* = 38 (%)	VEGF ≥ 365.27 pg/mL, *n* = 38 (%)	*p* Value
Rheumatoid arthritis	8 (21.1)	15 (39.5)	0.080
Spondyloarthropathies ^1^ Reactive arthritis	16 (42.1)	7 (18.4)	**0.025**
	9 (23.7)	3 (7.9)	0.059
Other autoimmune inflammatory diseases ^2^	4 (10.5)	6 (15.8)	0.497
Arthritis resolved	10 (26.3)	10 (26.3)	1.000

^1^ spondyloarthropathies ( reactive arthritis, axial or peripheral spondylarthritis, psoriatic arthritis); ^2^ other autoimmune inflammatory diseases (systemic lupus erythematosus, undifferentiated connective tissue disease, IgG 4 related disease); *p* significant if <0.05; values with statistical significance are bolded.

**Table 11:** Association between early undifferentiated arthritis study cohort outcomes and VEGF level.

UA Outcome	OR (95% CI)	*p* Value
Rheumatoid arthritis	0.999 (0.998–1.000)	0.075
Spondyloarthropathies	1.001 (0.998–1.004)	0.376
Other autoimmune inflammatory diseases	1.000 (0.998–1.001)	0.732
Arthritis resolved	1.000 (0.999–1.002)	0.513

OR, odds ratio; 95% CI, confidence interval; UA, undifferentiated arthritis; spondyloarthropathies ( reactive arthritis, axial or peripheral spondylarthritis, psoriatic arthritis); other autoimmune inflammatory diseases (systemic lupus erythematosus, undifferentiated connective tissue disease, IgG 4 related disease); *p* significant if <0.05.

## Data Availability

The original datasets are not publicly available due to data protection policies. The data presented in this study are available on scientific request from the corresponding author.
